# On-Chip Control over Polyelectrolyte–Surfactant Complexation in Nonequilibrium Microfluidic Confinement

**DOI:** 10.3390/polym14194109

**Published:** 2022-09-30

**Authors:** Artem Bezrukov, Yury Galyametdinov

**Affiliations:** Department of Physical and Colloid Chemistry, Kazan National Research Technological University, Kazan 420015, Russia

**Keywords:** microfluidics, association, polyelectrolyte–surfactant interactions, aggregation, phase behavior, convection, diffusion

## Abstract

The goal of this work is to classify and quantify the factors that govern polyelectrolyte–surfactant complexation in microfluidic confinement and optimize the designs and operating modes of microfluidic reactors to offer additional advantages over the macroscopic synthesis of such complexes. We analyze and solve a system of governing convection–diffusion–reaction equations to conveniently represent these factors or their combinations as dimensionless similarity criteria. We discuss how these factors contribute to the on-chip control of the reaction initiation, the complex product distribution in a microfluidic device, and the phase behavior of the confined reacting flows and experimentally verify the results in microchips. This approach allows for designing microfluidic devices and setting their operating modes to avoid undesirable clogging by reaction products, control the initiation of the complexation reaction, and produce polyelectrolyte–surfactant aggregates with a broader size range and reduced dispersity.

## 1. Introduction

Complexes made up of oppositely charged polymers and surfactants have attracted a sustainable interest in polymer science, nanotechnology, and biotechnology [1,2,3,4,5,6]. Polymers and surfactants are important building blocks of functional materials and nanoparticles [7,8,9,10,11]. Polyelectrolytes, surfactants, and their combinations are extensively used in many industries, including pharmacy, cosmetics, and medicine [2,7,8,12,13,14]. They experience high demand for their use in drug delivery applications [7,8,15,16,17], template synthesis of nanoparticles [18], oil recovery [19], and oil spill cleanup [20].

A remarkable feature of such systems is their sensitivity toward a variety of factors. We can control their association and phase behavior, volume properties, and surface activity by varying their structure [21,22], concentration and ratio of components [3,5,23], solvent [24], pH [25], low-molecular additives [3,5,25], etc. The influence of these factors on the behavior of polymer–surfactant complexes was extensively studied in macroscopic solutions. Their macroscopic associative and phase behavior is conveniently described by equilibrium parameters, such as the polymer–surfactant concentration ratio and the degree of surfactant binding by a macroion [3,5,25,26,27].

Recent developments in microfluidics offer new opportunities for the controlled fabrication of polyelectrolyte nanoparticles that can be used in drug delivery and biomedical applications [28,29,30,31,32,33,34]. Microfluidics offers unique conditions for the synthesis and modification of polymer–surfactant soft matter systems [28,35,36,37,38,39]. In contrast to macroscopic conditions, reacting confined microflows are nonequilibrium [40,41]. In nonequilibrium microchannel conditions, reaction kinetics becomes an important factor. The additional factors that may influence the association of polymers and surfactants in a microchannel are the convection and diffusion of reacting species [37,42,43,44], as well as a nonuniform profile and a pressure-driven flow [45,46]. On the other hand, these factors offer opportunities to perform the synthesis of nonequilibrium polymer–surfactant complexes that cannot be produced in macroscopic conditions [41].

To synthesize and modify polyelectrolyte–surfactant complexes in microfluidic confinement for nanotechnology or biomedical applications, we need to consider a whole set of these factors. Multiple publications focus on the synthesis of polymer nanoparticles in microchannels. These publications, however, mostly discuss the practical aspects of applying microfluidic approaches such as flow focusing or droplet microfluidics to produce polymer nanoparticles or complexes of polymers and surfactants [28,29,31,36,37,39,40,41,43]. As opposed to bulk conditions, a common picture of the competing microscopic and macroscopic factors that can exert influence on the polyelectrolyte–surfactant association in microchannels definitely requires a thorough analysis and finalization.

The goal of this work is to classify and optimize the physical and chemical factors that govern polyelectrolyte–surfactant complexation in microfluidic confinement and optimize the designs of microfluidic reactors for the synthesis of such complexes. To achieve this goal, we developed a model that characterizes the interactions of polymers and surfactants in a microchannel. We experimentally verified this model by performing the microfluidic complexation of oppositely charged polyelectrolytes and surfactants. Grouped into dimensionless parameters such as the Peclet number, these factors offer convenient tools to control the initiation of the complexation reaction, the phase behavior of the confined polymer–surfactant solutions, and the sizes of their associates.

## 2. Materials and Methods

### 2.1. Materials

Polyacrylic acid (PAA) was purchased from Polysciences, Inc. (Warrington, PA, USA). PAA molecular mass is 90,000 g/mol. The polymer is sold as a viscous liquid (25% aqueous solution) and was used as received. Polydiallyldimethylammonium chloride (PDADMAC) was purchased from Sigma Aldrich. The polymer is sold as a viscous liquid (20% aqueous solution) and was used as received. Cetyltrimethylammonium bromide (CTAB) and sodium dodecyl sulfate (SDS) were purchased from BDH Limited, Poole, England, and used as received. CTAB and SDS are sold as powders.

We selected these materials as model substances because the macroscopic association and phase behavior of such types of polyelectrolytes and surfactants are well-characterized [3,5,23,47]. We can find the CTAB and SDS diffusion coefficients in the literature [48] and estimate the polyelectrolyte–surfactant association dynamics and rate constants [25,49,50,51]. Thus, these substances are convenient for numerical simulations and ensure a smooth comparison of the experimental and numerical data.

Deionized water (18.2 MΩ-cm) was used for all the solutions. Before preparing the solutions, water was filtered with 0.2 µm Millipore PTFE filters.

Polydimethylsiloxane (PDMS) Sylgard 184 was purchased from Dow Corning (Midland, MI, USA) and used to fabricate microfluidic devices. It comes as a two-part elastomer kit (pre-polymer and the curing agent). SU-8 3050 photoresist (Microchem Corp., Westborough, MA, USA) was used to produce a mold for microfluidic chips.

### 2.2. Solutions

For both macroscopic and microfluidic experiments, the bulk samples of 1 g/L PAA and PDADMAC were produced from their initial solutions and allowed to dissolve overnight. Briefly, 50 mmol/L KBr was used as a background electrolyte. The concentration of monomers in 1 g/L PAA solution was 1.4 × 10^−2^ mol/L. The bulk samples of 1.4 × 10^−2^ mol/L CTAB were produced by dissolving the dry surfactant in deionized water. The respective concentration of monomers in 1 g/L PDADMAC solution was 6.1 × 10^−3^ mol/L. The bulk samples of 6.1 × 10^−3^ mol/L SDS were produced by dissolving the dry surfactant in deionized water.

For macroscopic experiments, PAA-CTAB and PDADMAC-SDS solutions were combined in different ratios by volume to provide polymer–surfactant compositions with the surfactant-to-polymer molar ratios varying in the 0–2.0 range.

The PAA and CTAB solutions were infused into the microfluidic devices using Harward Apparatus 2000 syringe pumps. The flow rates of the polymer and surfactant solutions and the solvent varied in the range of 0.2–50 µL/min. The duration of all the microfluidic experiments was 15 min unless otherwise specified.

To collect the solutions downstream of single-output or multi-output microfluidic devices, we connected PTFE tubes of identical lengths (10 cm) and internal diameters that fit the needle tips inserted into microchip outputs (20 G-type needles, 0.9 mm diameter). These identical tubes provided a uniform release of solutions from multiple outputs. The collecting reservoirs were either 2 mL glass vials or disposable polystyrene Malvern Zetasizer microcuvettes with an analytical volume of 40 μL. Such cuvettes allow for the collection of sufficient amounts of samples for the dynamic light scattering (DLS) analysis within microfluidic experiment times of 15 min.

### 2.3. Methods

The hydrodynamic diameters and polydispersity indices of PAA and PDADMAC macromolecules, PAA-CTAB, and PDADMAC-SDS complexes synthesized in macroscopic and microfluidic solutions were measured using the Malvern Zetasizer Nano ZSP light scattering system. Hydrodynamic sizes and the diffusion coefficients corresponding to the maximum of the number-average size distribution of the particles were used in the simulations and experiments. PDI is the polydispersity index of aggregates taken from the Malvern Zetasizer Nano software report. The PDI range is [0;1]. PDI values are lower for monodisperse particles and higher for polydisperse particles. All the DLS measurements were repeated at least three times to obtain reproducible results. The reported diameters of the particles measured using DLS correspond to the maximums of the number-average distribution curves.

We used an Akvilon pH-410 pH meter with a combined pH electrode to measure the pH of a macroscopic PAA solution.

The images were recorded on a Levenhuk D320 optical microscope. The microchannels and precipitated PAA-CTAB aggregates were imaged at 10× magnification using a Levenhuk M1400 Plus camera with a resolution of 0.27 µm/pixel.

The convection–diffusion–reaction equations for reacting polyelectrolyte–surfactant flows were solved using the MATLAB 2021a software with the Partial Differential Equations Toolbox. The diffusion coefficients of CTAB and SDS ions were obtained from [48]. For the simulations, the value of the polyelectrolyte–surfactant association rate constant was set to 10^4^ L·mol^−1^·s^−1^.

### 2.4. Device Fabrication

The microfluidic devices were fabricated using standard photolithography techniques [52]. SU-8 photoresist and a transparency photomask with the negative image of a microchip were used to produce a 100 µm thick mold of microfluidic chips on top of a 3-inch silicon wafer. Sylgard 184 PDMS pre-polymer was mixed with a curing agent, poured over the mold, and allowed to cure for 4 h in a 60 °C oven. Once cured, PDMS was peeled off the mold and bonded to a flat PDMS slab via plasma treatment. The PDMS device was then heated in an oven at 180 °C for 1 h to finalize the bonding of the two polymer layers.

## 3. Results

### 3.1. Bulk Characterization of Polymer–Surfactant Systems

Depending on the nature and characteristics of the reacting species, we can observe multiple scenarios of macroscopic polyelectrolyte–surfactant complexation and phase behavior [3]. The unfolding of such scenarios can be conveniently described by the dimensionless number Z, which is the ratio of the molar surfactant concentration to the concentration of monomers in a polymer–surfactant solution [3]. Before the microfluidic experiments, we performed a series of bulk tests to characterize the purchased polyelectrolytes and surfactants, verify their complexation scenario, and determine the threshold Z values that correspond to the transitions between their phase states.

**Figure 1:**
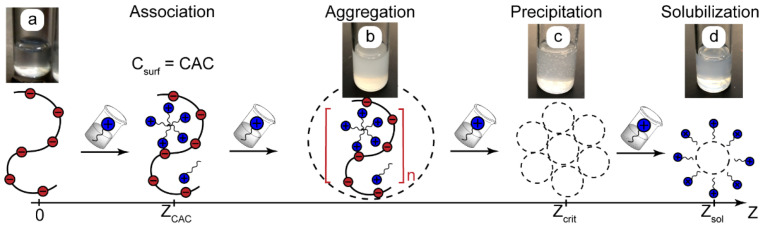
A possible scenario of complexation and phase behavior in solutions of oppositely charged polymers and surfactants depending on the surfactant-to-polymer ratio Z: (**a**)—polymer solutions, (**b**)—aggregation, (**c**)—precipitation, (**d**)—solubilization of insoluble aggregates. CAC is the critical association concentration. Z_crit_ is the value of Z corresponding to precipitation. Red dots are polyelectrolyte binding centers, blue dots are surfactant head groups, and n is the degree of aggregation. The photos are PAA (Z = 0) and PAA-CTAB solutions.

Figure 1 shows a scenario of the association and phase behavior of macroscopic systems made up of oppositely charged polyelectrolytes and surfactants depending on the Z ratio.

The critical association concentration (CAC) is a parameter that is analogous to the critical micellar concentration (CMC) of surfactant solutions. When the surfactant concentration reaches CAC, surfactants form micelle-like aggregates on a macromolecular matrix due to a hydrophobic effect (cooperative binding). The binding isotherms of such systems [3,5] show that, above CAC, macrochains bind a major part of the added surfactant.

The cooperative binding of surfactant ions above CAC may strongly reduce the solubility of macromolecules and initiate their aggregation (turbid solutions, Figure 1b) followed by precipitation at sufficiently high concentrations of added surfactant (Figure 1c). Such associative phase separation is described in the literature for different mixtures of polyelectrolytes with oppositely charged ionic surfactants in general [3,5], for the reagents used in this work [25,53], and also in our previous experiments with polyelectrolyte–surfactant systems [27]. For convenience, we can describe the precipitation threshold as the critical polymer-to-surfactant ratio Z_crit_ [27]. Finally, additional surfactant molecules may solubilize the precipitates, so we return to a homogeneous system (Figure 1d).

The scenario shown in Figure 1 was verified and confirmed for the reaction pairs used in this work. The bulk characterization of PAA-CTAB solutions showed that they became turbid when Z << 0.1 and formed precipitates at Z_crit_ < 0.1. In the range of Z = 0.1–0.9, the solutions remained turbid, and dynamic light scattering showed large aggregating particles with a diameter of above 500 nm. At Z > 1, the precipitates dissolved, and the solutions contained PAA-CTAB aggregates of about 100 nm in diameter. The hydrodynamic number-average diameter of individual PAA macromolecules was about 10–15 nm.

These results agree with the literature data that indicate a similar behavior of PAA-CTAB systems [25,53]. Under such conditions, PAA is a weakly charged polyelectrolyte. The small amounts of added surfactants are supposed to reduce PAA’s solubility and initiate precipitation. According to our potentiometry data, the pH of the PAA solution was 3.3, which agrees with [25], reporting pH = 3.4 for the same solution of a similar PAA sample and finding the degree of PAA ionization to be ≈3%.

The PDADMAC-SDS solutions became turbid at Z ≈ 0.1 and precipitated at Z_crit_ ≈ 0.5. Within this range, the aggregates grew in size, from approximately 50 nm to 200 nm. The precipitates dissolved, and the solutions returned to a homogeneous state at Z ≈ 1.5. At Z > 1.5, the precipitates dissolved, and the solutions contained the PDADMAC-SDS aggregates of about 100–150 nm in diameter. The hydrodynamic number-average diameter of individual PDADMAC macromolecules was about 15 nm.

For microfluidic experiments, we selected the PAA and CTAB solutions that provide Z = 1 and the PDADMAC and SDS solutions that provide Z = 0.1 upon mixing in a microchip. In our bulk experiments, the solutions of the resulting complexes were homogeneous. They are expected not to clog a microfluidic chip. They contain nanoscale or sub-microscale complexes that are sensitive to different concentrations of the reagents. Microfluidic tools are supposed to provide additional factors for controlling the properties of such complexes by creating variable concentration gradients with different chip geometries and flow rates.

### 3.2. Synthesis of Polymer–Surfactant Complexes in Standard Microfluidic Chips with Y-Junction and Flow-Focusing Geometries

To synthesize polyelectrolyte–surfactant complexes in microchannels, we followed a basic assumption that the complexation processes shown in Figure 1 are the same both in macroscopic conditions and inside a microfluidic chip. The widths of the microchannels in our microchips (100–300 μm) were several orders of magnitude larger than the sizes of the reacting species (nanometers) and complexes (dozens or hundreds of nanometers). A microscale microfluidic confinement is, therefore, supposed to exert an insignificant influence on the thermodynamics and kinetics of intermolecular interactions that are responsible for the binding of surfactant ions by polyelectrolyte ionogenic groups.

To synthesize the polymer–surfactant complexes in a microfluidic confinement, we selected two designs of reactors: Y-type chips and flow-focusing chips. Such devices are standard and most widely used for the microfluidic synthesis of organized media based on polymers and surfactants [31,32,34,35,37,38,39]. The reacting flows in such chips are laminar and parallel due to very low Reynolds numbers (Re ≈ 1–10) [42,43,44].

**Figure 2:**
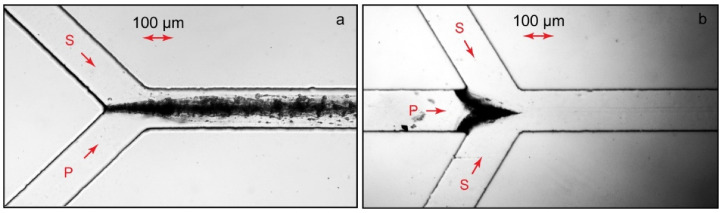
Microfluidic synthesis of PAA-CTAB complexes (Z = 1) in contacting flows of a Y-junction chip (**a**) and via flow focusing (**b**). P—polyelectrolyte, S—surfactant. Polyelectrolyte flow rates are 10 µL/min (**a**) and 1 µL/min (**b**). Surfactant flow rates are 10 µL/min. Chip lengths L = 15 mm and widths W = 200 μm. Duration of the experiments: 15 min.

Figure 2 demonstrates the microscopic photos of PAA-CTB complexes in such chips.

In a Y-type chip (Figure 2a), the solutions of reagents are fed through different inputs to contact the main channel after the junction. In such reactors, equal or comparable flow rates of reacting solutions are set [44]. A flow-focusing chip is designed with three inputs (Figure 2b): One of the reagents is fed through the central input. Another reagent is fed by side flows. The side flow rates are set to be much higher than those of the central one. The reaction zone is, therefore, limited by a very thin central flow and is supposed to be completed within the residence time of the reacting species in microchannels, even for slowly diffusing colloid and polymer particles [39,43].

In our experiments, therefore, we set equal flow rates of polymer and surfactant solutions (10 µL/min) in Y-type chips and a higher surfactant flow rate (10 µL/min on each side) with a lower central polymer flow rate (1 µL/min). The total flow rates in the main channel were 20 µL/min (Figure 2a) and 21 µL/min (Figure 2b). Such flow rates were set to provide the residence time of the reacting species in the main channels of the chips as ≈1 s. In our macroscopic experiments, such a time period was sufficient to observe substantial changes in the phase behavior of polymer solutions upon the addition of surfactants that indicate a complexation reaction.

Figure 2 shows that the phase behavior of the confined polyelectrolyte–surfactant solutions was different from that of the bulk experiment even at the microscale. Although the respective bulk solutions of complexes were homogeneous, microfluidic solutions with the same values of Z underwent phase separation.

The samples collected at the outputs were, however, homogeneous. According to DLS, the size and dispersity of the complexes were similar to those originally synthesized in bulk: hydrodynamic diameter D ≈ 100 nm and polydispersity index PDI = 0.25–0.3.

A set of additional microfluidic experiments was performed in various conditions with PAA-CTAB and PDADMAC-SDS systems. The experiments were repeated with various chip designs and operating modes (Y-chips, Ψ-chips, width = 100–300 µm, length = 15–30 mm, Q_Polymer_ = Q_Surfactant_ = 0.2–50 µL/min, and Z = 0.1–1.5) and always resulted in precipitation and chip clogging, the same as shown in Figure 2. We revealed no significant effect of any potential microfluidic control factors (such as microchannel lengths and widths or the flow rates of the reagents) on the phase behavior of polyelectrolyte–surfactant solutions and the sizes of their complexes.

### 3.3. Modeling and Optimization of Microfluidic Reactors for Polyelectrolyte–Surfactant Complexation

**Figure 3:**
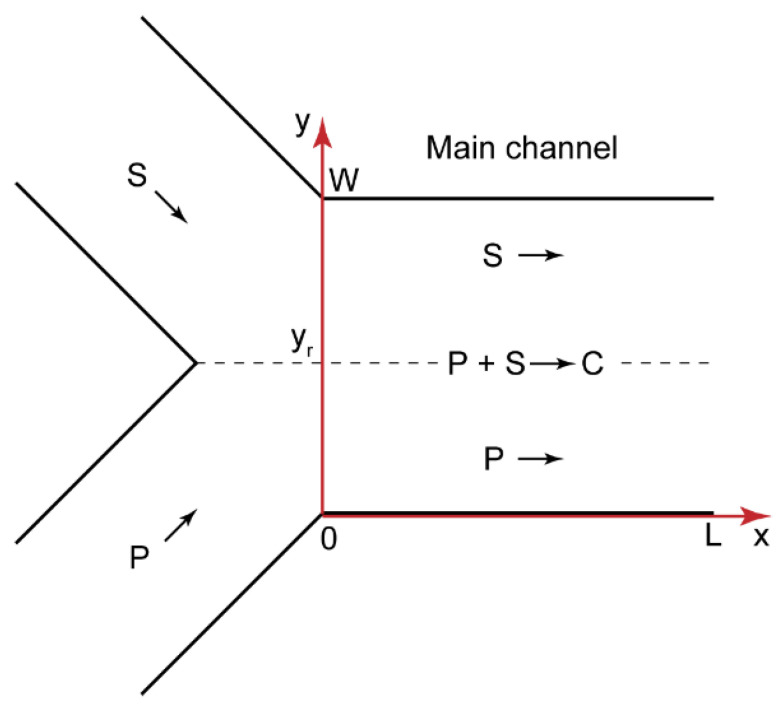
Geometry of a Y-type microfluidic chip with the length L and the width W; P—polyelectrolyte, S—surfactant, C—complex; y_r_ is the radial coordinate of the reaction front.

Precipitation in microchannels is a negative effect, which results in the clogging and failure of microchips. To reveal and avoid those factors that govern precipitation in confined solutions, we proceeded to develop a model of polyelectrolyte–surfactant interactions in confinement. Figure 3 shows the geometry of a microchip corresponding to Figure 2a. The reacting flows were laminar (for the performed microfluidic experiments, the Reynolds number Re < 10), so the parallel flows of the reacting species formed after the junction. The reaction occurred at the contact line of these polymer and surfactant flows.

The interaction of surfactant molecules with monomer binding sites on a macromolecule can be approximated using a second-order reaction [3,50,51]:

(1) P+S↔C

where P is polyelectrolyte, S is the surfactant, and C is their complex.

In the microchannel shown in Figure 3, reaction (1) can be generally described by a system of convection–diffusion–reaction equations for the polymer, surfactant, and their interaction product [44,54]:

(2) $$\{\begin{array}{c} U\left(y\right){\left[P\right]}_{x}^{\prime }=D_{P}{\left[P\right]}_{\mathrm{yy}}^{\prime \prime }-k_{f}\left[P\right]\left[S\right]+k_{r}\left[C\right]\\ U\left(y\right){\left[S\right]}_{x}^{\prime }=D_{S}{\left[S\right]}_{\mathrm{yy}}^{\prime \prime }-k_{f}\left[P\right]\left[S\right]+k_{r}\left[C\right]\\ U\left(y\right){\left[C\right]}_{x}^{\prime }=D_{C}{\left[C\right]}_{\mathrm{yy}}^{\prime \prime }+k_{f}\left[P\right]\left[S\right]-k_{r}\left[C\right] \end{array}$$

where [P] is the molar concentration of monomer binding sites in the polyelectrolyte solution, [S] is the surfactant concentration, and [C] is the molar concentration of the monomer binding sites that bound surfactant ions; D_P_, D_S_, and D_C_ are the diffusion coefficients of polymer macromolecules, surfactant molecules, and polymer–surfactant complexes, respectively; x and y are the axial and radial coordinates; U(y) is the flow velocity; k_f_ and k_r_ are the forward and reverse reaction rate constants, respectively.

The zero-flow boundary conditions for the microchannel walls [44] of the polymer are found as follows:

(3) $${\left[P\right]}_{y}^{\prime }{|}_{y=0;y=W}=0$$

This is the same for surfactant S and complex C.

The boundary conditions for the polymer, surfactant, and product concentrations at the junction of the input flows (x = 0) [44] are:

(4) $$\{\begin{array}{c} \left[P\right]\left(x=0,y\right)=\{\begin{array}{c} {\left[P\right]}^{0},y\;\leq y_{r}\\ 0,y>y_{r}\; \end{array}\\ \left[S\right]\left(x=0,y\right)=\{\begin{array}{c} 0,y<y_{r}\\ {\left[S\right]}^{0},y\geq y_{r}\; \end{array}\\ \left[C\right]\left(x=0,y\right)=0\;\;\;\;\;\;\;\;\;\;\;\;\;\;\;\;\;\; \end{array}$$

where [P]^0^ and [S]^0^ are the initial concentrations of the polymer and surfactant, respectively.

Equations (2)–(4) constitute the mathematical model of the polymer–surfactant complexation reaction occurring in the microchip shown in Figure 3. This model considers two dimensions (length and width) without the height of the main channel and neglects the wall interaction effects. A 2D approach was successfully applied in multiple works on microfluidic diffusion and reaction systems [42,44,46,54,55,56]. The laminar flows of the reacting solutions in such systems are strictly parallel to the “x” axis in the main channel due to a very low Reynolds number. The input flows in our microfluidic devices were steady-state. The diffusion of the reacting species along the microchannel length (the “x” axis) and height (the “z” axis) could, therefore, be neglected. The system behavior was only governed by a cross-stream diffusion of the reacting species.

A limitation of this 2D model is, however, that we did not consider the dependence of the flow velocity on the microchannel height (the vertical dimension). The previous simulations of parallel axial microfluidic flows in 3D [57,58] revealed that the concentration profiles of the diffusing species are symmetrical along the microchannel height. A more intensive cross-stream diffusive migration of the reacting species can be expected near the microchannel’s top and bottom walls due to the reduced flow velocities in these zones. This may lead to a slight additional product accumulation but is not supposed to significantly change the overall product concentrations at the main channel output.

**Figure 4:**
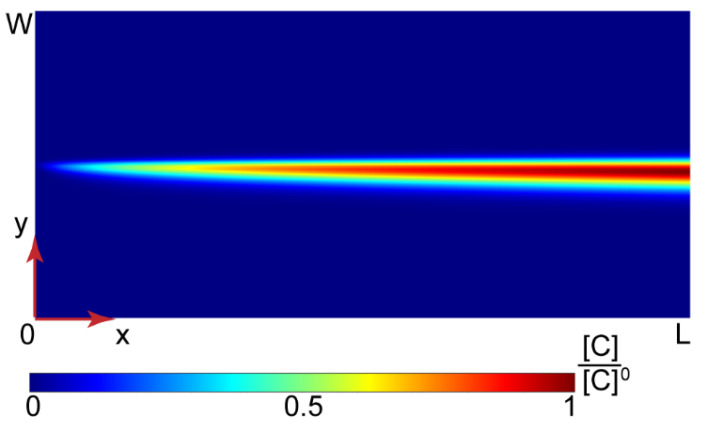
Numerical solution for the PAA-CTAB complexation in a Y-type microfluidic chip (L = 15 mm and W = 200 μm). All the input flow rates are 10 µL/min.

Equation (2) with the boundary conditions represented by Equations (3) and (4) were transformed into a dimensionless form and solved numerically. More details about this mathematical model, the dimensional analysis, and numerical simulations are provided in the Supplementary Materials and Figures S1 and S2. Figure 4 shows the solution for the PAA-CTAB complexation product obtained in MATLAB.

Figure 4 demonstrates that the complex product accumulated in the central part of a microchannel where the reaction occurred. Further simulations of Y-type chips with widths = 100–300 µm, lengths = 15–30 mm, and flow rates of the reagents in the range of 0.2 = 50 µL/min also predicted high product concentrations in the central part of the main channel, the same as in Figure 4.

In a microchannel, therefore, surfactant ions are likely to preferably interact with the binding sites on polyelectrolyte macromolecules available in a narrow reaction zone due to cooperative binding. The bound surfactant molecules reduce the solubility of polymer chains and may initiate their aggregation and precipitation in a narrow reaction zone before a uniform equilibrium concentration distribution of reacting species is reached. Precipitation conditions, therefore, can be achieved in the contacting microscale flows even if the initial solutions are prepared to provide Z < Z_crit_.

Therefore, traditional microfluidic reactor geometries (Y-junction and flow-focusing chips) with the contacting flows of reagents may be unsuitable for the synthesis of polymer–surfactant complexes that tend to precipitate in bulk.

**Figure 5:**
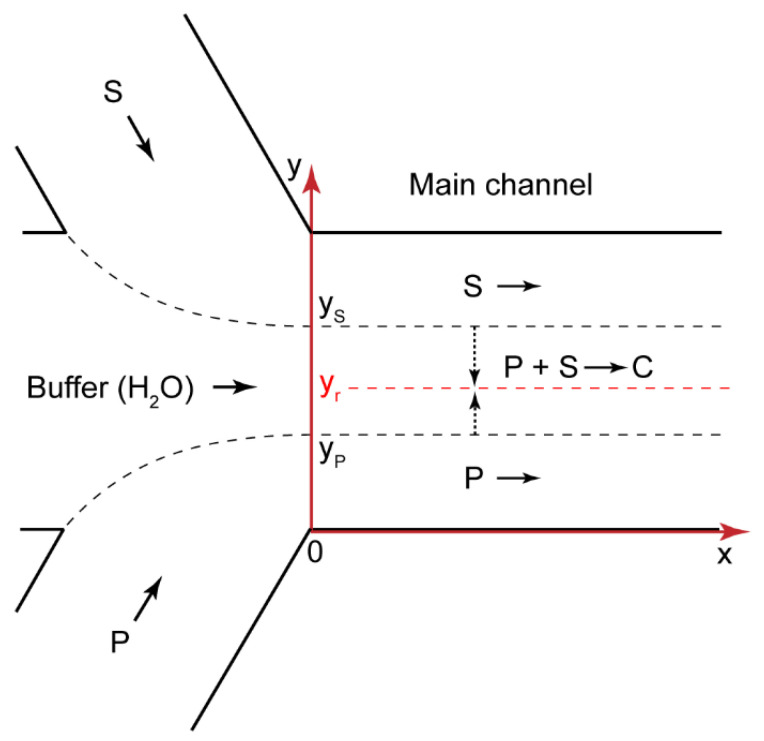
Geometry of a Ψ-type microfluidic chip. P—polyelectrolyte, S—surfactant, C—complex; y_r_ is the radial coordinate of the reaction front; y_P_ and y_S_ are polymer and surfactant flow boundaries, respectively.

To avoid the precipitation initiated by the contacting of polymer and surfactant flows, we redesigned the microfluidic reactor by introducing a central flow of a buffer. Figure 5 shows the resulting three-input (Ψ-type) microchip geometry.

**Figure 6:**
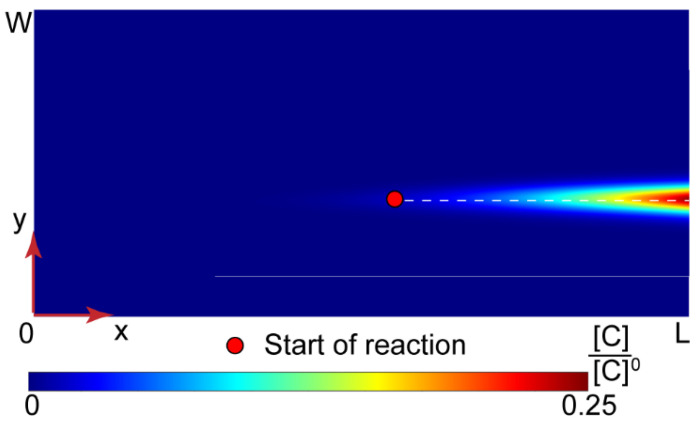
Numerical solution for the PAA-CTAB complexation in a Ψ-type microfluidic chip (L = 15 mm and W = 200 μm). All the input flow rates are 10 µL/min.

Equation (2) was solved for Figure 5 geometry and boundary conditions. The numerical solution for the product is demonstrated in Figure 6.

With the central solvent flow, the reaction did not start immediately after the junction of inputs, and the product concentrations in the main channel were much lower than those in Y-shape chips.

**Figure 7:**
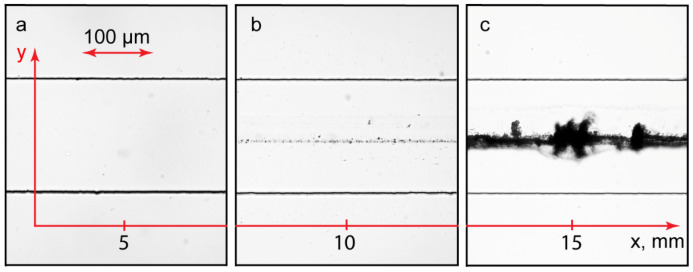
PAA-CTAB complexation reaction in a Ψ-type microfluidic chip (L = 15 mm and W = 200 μm). Distance from the junction of inputs, mm: 5 (**a**), 10 (**b**), and 15 (**c**). All the input flow rates are 10 µL/min. Duration of the experiment: 15 min.

To experimentally verify the approach illustrated in Figure 5 and Figure 6, we fabricated a Ψ-type microfluidic reactor. The results of the microfluidic experiments are shown in Figure 7.

There was a correlation between the numerically predicted product concentration and the accumulation of precipitates in the main channel. Precipitation was more intensive near the channel output (Figure 7c) and reduced upon approaching the junction of the inputs (Figure 7b). Near the junction, the main channel was clean (Figure 7a). The samples taken from the output were homogeneous. The size and dispersity of the complexes were similar to those of their associates synthesized in bulk experiments. The same results were obtained for the PDADMAC-SDS system.

**Figure 8:**
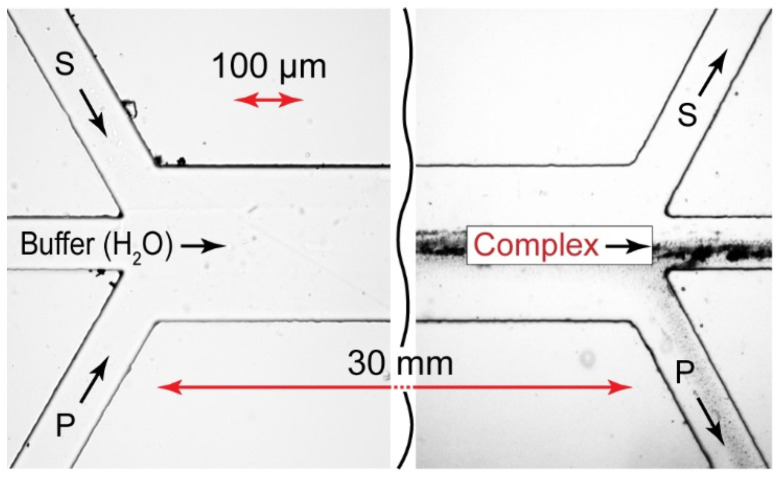
Ψ–Ψ-type microfluidic chip prototype. The testing reaction pair is PDADMAC + SDS. All the flow rates are 10 µL/min. Samples are taken from the central output. Duration of the experiment: 15 min.

The microfluidic reaction fronts in Y-type and Ψ-type microfluidic chips produced complexes with relatively high local concentrations. According to our numerical simulations (Figure 4 and Figure 6), however, the product yield in confinement was only 3–5%. The reaction is supposed to complete in macroscopic conditions after the reagents left the main channel. To separate the microfluidic complexation products from the unreacted species, we implemented the multi-output approach discussed in [41]. Figure 8 shows the prototype of the microfluidic chip (a Ψ–Ψ chip) and the respective experiment.

Such a microchip design allows for the collection of nonequilibrium complexation products from the central output, while precipitation is kept at a tolerable minimum.

### 3.4. Selection of Governing Factors for Confined Polyelectrolyte–Surfactant Complexation

The next stage of this work focused on identifying and classifying the microfluidic factors that may control polyelectrolyte–surfactant complexation in confinement and allow for the optimization of the geometry and operation of Ψ–Ψ-type and Ψ–Ψ-type chips. The starting point was to analyze the variables in Equations (2)–(4). According to these equations, the behavior of a polyelectrolyte–surfactant reaction system in a microfluidic chip depends on many more factors than in bulk:

- The concentrations of polymer [P], surfactant [S], and complex [C];
- The diffusivities of polymer macromolecules D_P_, surfactant molecules D_S_, and polymer–surfactant complexes D_C_;
- The rate constants of the association reaction k_f_ and the reverse reaction k_r_;
- Microchannel geometry set by its width W and length L;
- The polymer and surfactant flow rates Q_P_ and Q_S_, which set the flow velocity U in the main channel.

To reduce the number of these factors and enhance their applicability, we grouped them into similarity criteria by performing a dimensional analysis of Equations (2)–(4). The details of this analysis are provided in the Supplementary Materials.

The system of Equation (2) was modified into a dimensionless form by introducing dimensionless coordinates x* = x/L and y* = y/W and polymer concentrations [P]* = [P]/[P]^0^, (the same dimensionless concentrations were introduced for the surfactants and complexes).

Equation (5) shows the results of the dimensional analysis:

(5) $$\{\begin{array}{c} \beta \frac{\mathrm{Pe}}{L_{N}}{\left[P\right]}_{x}^{{*}^{\prime }}={\left[P\right]}_{\mathrm{yy}}^{{*}^{\prime }}-Da\left(Z{\left[P\right]}^{*}{\left[S\right]}^{*}-Z_{\mathrm{CAC}}{\left[C\right]}^{*}\right)\\ \beta \frac{\mathrm{Pe}}{L_{N}}{\left[S\right]}_{x}^{{*}^{\prime }}=D_{N}{\left[P\right]}_{\mathrm{yy}}^{{*}^{\prime \prime }}-Da\left({\left[P\right]}^{*}{\left[S\right]}^{*}-\frac{Z_{\mathrm{CAC}}}{Z}{\left[C\right]}^{*}\right)\\ \beta \frac{\mathrm{Pe}}{L_{N}}{\left[S\right]}_{x}^{{*}^{\prime }}=D_{\mathrm{CN}}{\left[P\right]}_{\mathrm{yy}}^{{*}^{\prime \prime }}+Da\left(Z{\left[P\right]}^{*}{\left[S\right]}^{*}-Z_{\mathrm{CAC}}{\left[C\right]}^{*}\right) \end{array}$$

where the function β represents a parabolic flow velocity profile in the main channel. For the coordinates in Figure 5, β = 3/2(1 − (2y* − 1)^2^). $D_{N}={\;D}_{S}/D_{P}$ and $D_{\mathrm{CN}}={\;D}_{C}/D_{P}$ are the dimensionless numbers that represent the diffusivities of surfactant D_S_ and product D_P_ normalized by the diffusivity of the polymer.

The boundary conditions at the junction of inputs in Ψ-type chips provide another similarity criterion. The initiation of the complexation reaction requires the diffusion of the reacting species to the reaction zone in a buffer flow. In turn, the diffusion pathways are governed by the width of the buffer flow. The buffer flow width can be represented as the ratio of the solvent flow rate Q_S_ to the sum of all the flow rates: Q_N_ = Q_S_/∑Q_i_.

Table 1 summarizes the dimensionless numbers and subdivides them into several groups. For convenience, the similarity criteria in all the equations depend on polymer concentration [P]^0^ and diffusivity D_P_.

The high Peclet number demonstrates that convection prevails diffusion, and the equilibrium concentrations of the reacting species are not reached before the microchannel output. A reacting polymer–surfactant system in a microchannel is nonequilibrium, and the equilibrium parameters Z and CAC are not suitable for characterizing the association and phase behavior of such systems in a microchannel.

The Damköhler number Da >> 1, so the reaction rate was much higher than the diffusion rate, and we can assume without a significant error that the polymer and surfactant reacted almost instantaneously at the point where they interacted via diffusion [59]. This agrees with the further numerical simulations that we performed for various association rate constants (10^2^–10^7^) and demonstrated that the product concentration distribution remained virtually the same. These additional results are provided in the Supplementary Materials (Figure S3).

We could, therefore, use the approximate values of the rate constants and neglect the “Reaction” group parameters in Table 1 to model the polyelectrolyte–surfactant complexation in a microchannel.

The low diffusion coefficient D_C_ of the product favored its accumulation in the reaction zone and did not exert a significant influence on the interactions of the incoming polymer and surfactant molecules. This also agrees with our numerical simulations performed for various D_C_ values (0.1–1000).

Therefore, we can characterize the polymer–surfactant complexation in a microchannel in typical microchip operating conditions with only 4 governing similarity criteria instead of the initial 12-dimensional parameters:

- Pe and Q_N_ represent the microchip’s operating mode;
- L_N_ represents the microchip’s geometry;
- D_N_ represents the properties of the reagents.

These parameters can be grouped to predict the reaction’s initiation conditions in a Ψ-type microchip. According to Figure 5, the reaction occurs if there is sufficient time for the reagents to diffuse to the reaction site. The initiation of a polyelectrolyte–surfactant complexation reaction in the main channel depends, therefore, on the diffusivities of the reagents, microchannel length and width, and flow rates. Equation (6) formalizes the contribution of all these factors as the combination of similarity criteria for symmetrical polyelectrolyte and surfactant flows:

(6) $${\left(\frac{\mathrm{Pe}}{L_{N}}\right)}_{\mathrm{in}}=4{\left(\frac{1+\sqrt{D_{N}}}{Q_{N}}\right)}^{2}$$

The details of Equation (6) derivation are provided in the Supplementary Materials.

The right part of Equation (6) depends only on the diffusivities of the reagents and the buffer flow width. The left part represents an integrated dimensionless number ${\left(\frac{\mathrm{Pe}}{L_{N}}\right)}_{\mathrm{in}}$, which evaluates the reaction initiation conditions for arbitrary microchannel lengths, widths, and flow rates.

Further simulations and experiments were performed for Ψ-type and Ψ–Ψ-type chips with widths = 100–300 µm, lengths = 15–30 mm, and flow rates 0.2 = 50 µL/min. The results were in good agreement with those found using Equation (6). With the experimental conditions providing $\frac{\mathrm{Pe}}{L_{N}}\geq {\left(\frac{\mathrm{Pe}}{L_{N}}\right)}_{\mathrm{in}}$, zero or limited precipitation was observed in the microchannels. Lowering $\frac{\mathrm{Pe}}{L_{N}}$ by either elongating or narrowing the main channel or reducing the flow rates resulted in the accumulation of precipitates.

### 3.5. Synthesis of Polyelectrolyte–Surfactant Complexes in Ψ-Type and Ψ–Ψ-Type Microchips

The numerical modeling and dimensional analysis stages allowed us to prioritize, group, and quantify the governing factors for the microfluidic polyelectrolyte–surfactant complexation and phase behavior. According to these results, we proposed two optimized designs of microfluidic reactors for the synthesis of polyelectrolyte–surfactant complexes: a Ψ-type chip and a Ψ–Ψ-type chip. We fabricated the microfluidic devices of these designs with varying lengths and widths of the main channel and performed polyelectrolyte–surfactant complexation experiments. Table 2 summarizes the characterization of the respective microfluidic samples with DLS.

For Ψ-type chips (no multiple outputs), the chip geometries and flow rates were selected to provide the Pe/L_N_ values above the reaction initiation threshold. As the reaction mostly occurred outside the main channel, the small chips with short (10–15 mm) and wide (300 μm) main channels were used for synthesis. Such compact chips provided easy control of the main channel under the microscope and produced no undesirable precipitates.

Ψ-type chips can be used as advanced pre-reactors that require significantly fewer efforts to synthesize a variety of polymer–surfactant aggregates from the same precursor solutions by changing the chip’s operating modes in a single operation cycle. The sizes (60–100 nm) and PDI (0.25–0.3) of these aggregates corresponded to their macroscopic analogues.

Polyelectrolyte–surfactant complexes are polydisperse aggregates. Malvern Zetasizer software reports the hydrodynamic diameter of these complexes as the maximum of their size distribution curve, while the polydispersity index (PDI) represents the width of this size distribution. Table 2 shows the mean positions of the size distribution maximums and the respective errors evaluated in our series of experiments.

For Ψ–Ψ-type microchips, the Pe/L_N_ values were varied and below the reaction threshold to initiate complexation reaction in the main channel while avoiding intensive precipitation. More details of precipitation time course in such conditions are provided in the Supplementary Materials, Figures S4–S6. For these experiments, we used the PDADMAC-SDS pair because it produced less abundant precipitates than the PAA-CTAB system. We fabricated Ψ–Ψ-type microchips with longer (30–100 mm) and narrower (100–200 μm) main channels. Such devices offer a higher threshold ${\left(\frac{\mathrm{Pe}}{L_{N}}\right)}_{\mathrm{in}}$ values than chips with shorter and wider main channels. Therefore, we can set higher flow rates to initiate the complexation reaction near the chip output and still minimize precipitation. Higher flow rates provide a faster sampling of the reaction products.

The size of the polymer–surfactant aggregates synthesized in Ψ–Ψ-type microchips (90–160 nm) differed from their macroscopic analogues (100 nm). In such conditions of synthesis, microfluidic complexes were less dispersed (PDI = 0.15–0.2) than their associates synthesized in bulk (PDI = 0.3). This agrees with the reports in the literature on more ordered complexation in microfluidic confinement than in bulk [35,39,43] due to a smooth diffusion-controlled mixing of the reagents.

## 4. Conclusions

In this work, we prioritized and quantified the governing factors of microfluidic polyelectrolyte–surfactant complexation. Due to the large diffusion coefficients of polymers and surfactants, their microfluidic reactive systems do not reach equilibrium. The reaction is diffusion-controlled, and we can use the approximate values of rate constants to model the polyelectrolyte–surfactant association. The equilibrium macroscopic complexation parameters lose their predictive power. The complexation process is governed by chip hydrodynamics, geometry, and diffusivities of the reagents. The combinations of these parameters as similarity criteria allow for the convenient prediction of reaction initiation conditions and the phase behavior of the complexes using arbitrary geometries and operating modes of microchips.

The microfluidic cooperative binding of surfactants by polymers occurs in extremely confined areas. The contacting flows of these reagents produce precipitates and clog channels even if the respective macroscopic systems are homogeneous. The Y-type and flow-focusing microchips are unsuitable for the synthesis of polymer–surfactant complexes that tend to precipitate in bulk.

We proposed two new designs of microchips specifically for polyelectrolyte–surfactant systems. The three-input chips with a central buffer flow minimize precipitation. As compared with the synthesis in bulk, such chips require significantly fewer efforts to produce a variety of polymer–surfactant aggregates from the same precursor solutions by changing the chip’s operating modes in a single operation cycle. Multiple input–output chips isolate nonequilibrium reaction products and synthesize less-dispersed complexes with a broader size range than those in macroscopic conditions. Such microfluidic circuits offer new options as structural components for laboratory-on-chip biopolymer analysis instruments or microchip devices for the fabrication of targeted drug delivery systems.

## Figures and Tables

**Table 1:** Dimensionless numbers for reactive microchannel flows of PAA-CTAB and PDADMAC-SDS.

Group	Dimensionless Number	Formula	Values
Macroscopic			
Reaction	Initial concentration ratio	Z = [S]^0^/[P]^0^	0.25–2
	Association concentration ratio	Z_CAC_ = CAC/[P]^0^	~0.01
Microfluidic			
Convection	Peclet number = convection rate/diffusion rate	Pe = UW/D_P_	~10^3^–10^5^
Reaction	Damköhler number = reaction rate/diffusion rate	$\mathrm{Da}=k_{f}{\left[P\right]}^{0}W^{2}/D_{P}$	~10^5^
Chip geometry	Normalized microchannel length	L_N_ = L/W	50–100
Diffusion	Normalized diffusion coefficients	D_N_ = D_S_/D_P_	~9–10
		D_CN_ = D_C_/D_P_	~0.1
Boundary conditions	Normalized buffer flow width	Q_N_ = Q_B_/∑Q_i_	0–1

**Table 2:** Hydrodynamic diameters and dispersities of microfluidic polymer–surfactant aggregates.

Reaction Pair	Chip	Dimensionless Parameters				Hydrodynamic Diameters and Dispersities			
		D_N_	Q_N_	Pe/L_N_	(Pe/L_N_)_in_	Microfluidic		Bulk (Reference)	
						D, nm	PDI	D, nm	PDI
PAA + CTAB	Ψ	10	0.33	1000	800	94 ± 20	0.25–0.3	104 ± 18	0.25–0.3
			0.5	500	350	82 ± 19		71 ± 15	
			0.67	300	200	61 ± 13		65 ± 17	
PDADMAC + SDS	Ψ–Ψ	9	0.33	300	800	164 ± 24	0.15–0.2	104 ± 18	
				500		121 ± 22			
				700		90 ± 15			

## Data Availability

Not applicable.

## Supplementary Materials

The following supporting information can be downloaded at: https://www.mdpi.com/article/10.3390/polym14194109/s1, Figure S1: Y-type microfluidic chip; P—polyelectrolyte, S—surfactant, C—complex; Figure S2: Geometry of a Ψ-type microfluidic chip. P—polyelectrolyte, S—surfactant, C—complex; Figure S3: Reaction front positions at different diffusion coefficients of the reaction product DC (1) and association rate constants kf (2); Figure S4: Accumulation of precipitates in the main channel of a Ψ-type chip (L = 15 mm and W = 200 μm). Distance from the junction of inputs: 10 mm. Input flowrates: polymer and surfactant: 10 µL/min, solvent: 40 µL/min; Figure S5: Accumulation of precipitates in the main channel of a Ψ-type chip (L = 15 mm and W = 200 μm). Distance from the junction of inputs: 10 mm. All the input flowrates are 5 µL/min; Figure S6: Accumulation of precipitates in the beginning of main channel of a Ψ-type chip (L = 15 mm and W = 200 μm). All the input flowrates are 1 µL/min.
