# Supplementary material for: On-Chip Control over Polyelectrolyte–Surfactant Complexation in Nonequilibrium Microfluidic Confinement
Source: Polymers (Basel). 2022 Sep 30;14(19):4109. doi: 10.3390/polym14194109 (PMC9571623; doi:10.3390/polym14194109)
Supplement: Supplementary file 1 [file polymers-14-04109-s001.zip › polymers-1909065-supplementary.pdf]

polymers

MDPI

On-Chip Control over Polyelectrolyte–Surfactant Complexation in Nonequilibrium Microfluidic Confinement

Artem Bezrukov and Yury Galyametdinov

S1. Mathematical Model for a Y-type Chip

We will start with the analysis of a microchip with 2 inputs (Fig. S1):

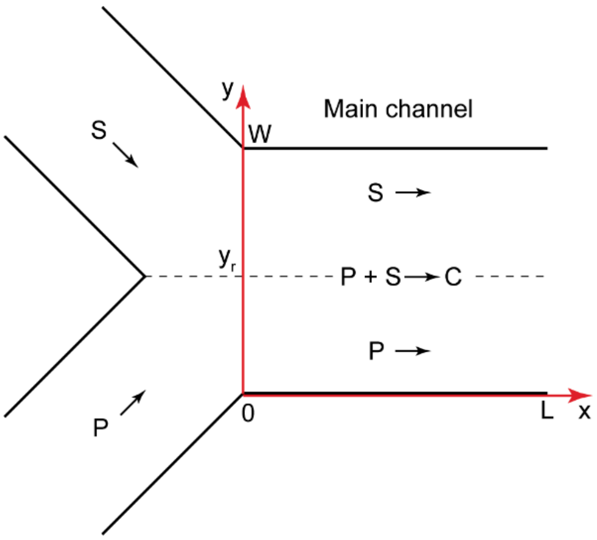

Figure S1. Y-type microfluidic chip; P – polyelectrolyte, S – surfactant, C – complex; yr is the radial coordinate of the reaction front.

Let us start with a general reaction equation:

$$P + S \leftrightarrow C \tag{1}$$

where P is the concentration of polyelectrolyte ionogenic groups, S is the concentration of surfactant ions, and C is the concentration of polyelectrolyte ionogenic groups with bound surfactant ions.

The rate law for polymer is:

$$[P]'_{\tau} = -k_f[P][S] + k_r[C] \tag{2}$$

where k_f is the association rate constant, k_f is the reverse reaction (complex dissociation) rate constant.

If such a reaction occurs in a microfluidic channel, we need to add the diffusion term $D_P\Delta[P]$:

$$[P]'_{\tau} = D_A\Delta[P] - k_f[P][S] + k_r[C] \tag{3}$$

and the convection term $\nabla[P]$:

$$[P]'_{\tau} + U(y)\nabla[P] = D_A\Delta[P] - k_f[P][S] + k_r[C] \tag{4}$$

where D_P is the diffusion coefficient of the polyelectrolyte ionogenic groups (equal to that of polymer macromolecules), U(y) is the flow velocity, ∇ is the Nabla operator Δ is the Laplacian operator, and x is the axial coordinate of a point in a microchannel.

To simplify this equation, consider only the axial convection of a pressure-driven laminar flow in a microchannel, only the radial diffusion of the reacting species and steady state conditions in the microchip [1,2]:

$$\mathrm{U(y)[P]'_x = D_A[P]''_{yy} - k_f[P][S] + k_r[C]} \tag{5}$$

where x and y are axial and radial coordinates in the main channel (Fig. S1), $[P]'_x$ and $[P]''_{yy}$ are the partial derivatives of polymer concentration in the main channel.

The Eq. (5) is a non-linear second-order partial differential equation with a reversible reaction as a source term.

Similar equations are derived for surfactant S and the reaction product C, so we obtain a system of partial differential equations that characterize behavior of a reacting polyelectrolyte-surfactant system in a microchannel shown in Fig. S1:

$$\begin{cases} \mathrm{U(y)[P]'_x = D_P[P]''_{yy} - k_f[P][S] + k_r[C]} \\ \mathrm{U(y)[S]'_x = D_S[S]''_{yy} - k_f[P][S] + k_r[C]} \\ \mathrm{U(y)[C]'_x = D_C[C]''_{yy} + k_f[P][S] - k_r[C]} \end{cases} \tag{6}$$

where [S] is the surfactant concentration, and [C] is the molar concentration of monomer binding sites that bound surfactant ions; D_S, and D_C are the diffusion coefficients of surfactant molecules and polymer-surfactant complexes.

The boundary conditions for the walls of the microchannel are derived from the assumption that the reaction species do not penetrate through them [1]. For polymer:

$$\begin{cases} \mathrm{[P]'_y(y = 0) = 0} \\ \mathrm{[P]'_y(y = W) = 0} \end{cases} \tag{7}$$

same for the surfactant S and complex C.

The boundary conditions for the junction of the input flows (x = 0) formalize that the concentration of a reagent is equal to the initial concentration in the incoming flow and is zero elsewhere, while the initial product concentration is zero [1]:

$$\begin{cases} \mathrm{[P](x = 0, y)} = \begin{cases} \mathrm{[P]^0, y \le y_r} \\ \mathrm{0, y > y_r} \end{cases} \\ \mathrm{[S](x = 0, y)} = \begin{cases} \mathrm{0, y < y_r} \\ \mathrm{[S]^0, y \ge y_r} \end{cases} \\ \mathrm{[C](x = 0, y) = 0} \end{cases} \tag{8}$$

S2.Dimensional Analysis of Convection-Diffusion-Reaction Equations

Let us introduce dimensionless parameters for the microchannel coordinates and concentrations of the reagents:

$$\mathrm{[P]^* = \frac{[P]}{[P]^0}, [S]^* = \frac{[S]}{[S]^0}, x^* = \frac{x}{L},} \text{ and } \mathrm{y^* = \frac{y}{W}} \tag{9}$$

where $[P]^0$ and $[S]^0$ are initial concentrations of the reagents, W and L are channel width and length, respectively.

From the reaction stoichiometry: $[C] = [P]^0 - [P]$, then:

$$\mathrm{\frac{[C]}{[P]^0} = 1 - [P]^*} \tag{10}$$

And we can introduce dimensionless concentration for a complexation reaction product:

$$\mathrm{[C]^* = \frac{[C]}{[P]^0}} \tag{11}$$

Consider the Hagen-Poiseuille parabolic flow profile in a rectangular microchannel. For the coordinates shown in Fig. S1:

$$U(y) = \frac{3}{2}U(1 - (2y^* - 1)^2) \quad (12)$$

Introducing the function $\beta = \frac{3}{2}(1 - (2y^* - 1)^2)$, we will finally get:

$$U(y) = \beta U \quad (13)$$

Let us transform the equation for polymer in the system of equations (6) into the dimensionless form:

$$\beta U \frac{[P]^0}{L}[P]^{*\prime}_x = D_P \frac{[P]^0}{W^2}[P]^{*\prime\prime}_{yy} - k_f[P]^0[S]^0[P]^*[S]^* + k_r[P]^0[C]^* \quad (14)$$

Divide this equation by $[P]^0$ and multiply by W^2/D_P:

$$\beta \frac{UW}{D_P}\frac{W}{L}[P]^{*\prime}_x = [P]^{*\prime\prime}_{yy} - \frac{W^2}{D_P}k_f[S]^0[P]^*[S]^* + \frac{W^2}{D_P}k_r[C]^* \quad (15)$$

Introduce the ratio Z of initial concentrations of the reagents S and P:

$$Z = \frac{[S]^0}{[P]^0} \quad (16)$$

Then:

$$\beta \frac{UW}{D_P}\frac{W}{L}[P]^{*\prime}_x = [P]^{*\prime\prime}_{yy} - \frac{W^2}{D_P}k_f Z[P]^0[P]^*[S]^* + \frac{W^2}{D_P}k_r[C]^* \quad (17)$$

Also note that:

$$k_r = k_f CAC \quad (18)$$

where CAC is the critical association concentration of surfactant and polyelectrolyte. Introduce the ratio Z_{CAC}:

$$Z_{CAC} = \frac{CAC}{[P]^0} \quad (19)$$

then:

$$k_r = k_f Z_{CAC}[P]^0 \quad (20)$$

The coefficients in the resulting dimensionless equation depend only on the properties of one reagent: polymer (diffusivity and initial concentration) and the association rate constant:

$$\beta \frac{UW}{D_P}\frac{W}{L}[P]^{*\prime}_x = [P]^{*\prime\prime}_{yy} - \frac{W^2}{D_P}k_f[P]^0(Z[P]^*[S]^* - Z_{CAC}[C]^*) \quad (21)$$

Several dimensionless similarity criteria appear in this equation:

Peclet number (the ratio of convection and diffusion rates):

$$Pe = \frac{UW}{D_P} \quad (22)$$

Normalized microchannel length (the ratio of microchannel length and width):

$$L_N = \frac{L}{W} \quad (23)$$

Damköhler number (the ratio of the characteristic time of diffusion to the characteristic time of reaction) for a second order direct reaction:

$$\mathrm{Da} = \left(\frac{W^2}{D_P}\right)/\left(\frac{1}{k_f[P]^0}\right) = \frac{W^2}{D_P}k_f[P]^0 \tag{24}$$

Then:

$$\beta\frac{\mathrm{Pe}}{L_N}[P]_x^{*\prime} = [P]_{yy}^{*\prime\prime} - \mathrm{Da}(Z[P]^*[S]^* - Z_{CAC}[C]^*) \tag{25}$$

All the dimensionless numbers are marked red.

The same dimensional analysis can be performed for the equations with the surfactant S and the product C. For surfactant:

$$\beta U\frac{[S]^0}{L}[S]_x^{*\prime} = D_S\frac{[S]^0}{W^2}[S]_{yy}^{*\prime\prime} - k_f[P]^0[S]^0[P]^*[S]^* + k_r[P]^0[C]^* \tag{26}$$

To use the same dimensionless numbers calculated for polymer P with the convection-diffusion-reaction equation for the surfactant S, let us introduce the dimensionless ratio of their diffusion coefficients $D_N = \frac{D_S}{D_P}$.

Divide the Eq. (26) by $[S]^0$ and multiply by W^2/D_P:

$$\beta\frac{UW}{D_P}\frac{W}{L}[S]_x^{*\prime} = \frac{D_S}{D_P}[S]_{yy}^{*\prime\prime} - \frac{W^2}{D_P}k_f[P]^0[P]^*[S]^* + \frac{W^2}{D_P}k_r\frac{[P]^0}{[S]^0}[C]^* \tag{27}$$

Modify Eq. (27) by substituting $\frac{[P]^0}{[S]^0} = \frac{1}{Z}$, $k_r = k_f Z_{CAC}[P]^0$ and $D_N = \frac{D_S}{D_P}$:

$$\beta\frac{UW}{D_P}\frac{W}{L}[S]_x^{*\prime} = D_N[S]_{yy}^{*\prime\prime} - \frac{W^2}{D_S}k_f[P]^0\left([P]^*[S]^* + \frac{Z_{CAC}}{Z}[C]^*\right) \tag{28}$$

Finally:

$$\beta\frac{\mathrm{Pe}}{L_N}[S]_x^{*\prime} = D_N[S]_{yy}^{*\prime\prime} - \mathrm{Da}\left([P]^*[S]^* - \frac{Z_{CAC}}{Z}[C]^*\right. \tag{29}$$

All the dimensionless numbers are marked red.

Dimensionless analysis of the equation for the product C:

$$\beta U\frac{[P]^0}{L}[C]_x^{*\prime} = D_C\frac{[P]^0}{W^2}[C]_{yy}^{*\prime\prime} + k_f[P]^0[S]^0[P]^*[S]^* - k_r[P]^0[C]^* \tag{30}$$

To use the same dimensionless numbers calculated for polymer P with the convection-diffusion-reaction equations for the product C, let us introduce the dimensionless ratio of their diffusion coefficients $D_{CN} = \frac{D_C}{D_P}$.

Divide the Eq. (30) by $[P]^0$ and multiply by W^2/D_P:

$$\beta\frac{UW}{D_P}\frac{W}{L}[C]_x^{*\prime} = \frac{D_C}{D_P}[S]_{yy}^{*\prime\prime} + \frac{W^2}{D_P}k_f[S]^0[P]^*[S]^* - \frac{W^2}{D_P}k_r[C]^* \tag{31}$$

Modify Eq. (27) by substituting $[S]^0 = Z[P]^0$, $k_r = k_f Z_{CAC}[P]^0$ and $D_{CN} = \frac{D_C}{D_P}$:

$$\beta\frac{UW}{D_P}\frac{W}{L}[S]_x^{*\prime} = D_{CN}[S]_{yy}^{*\prime\prime} + \frac{W^2}{D_S}k_f[P]^0(Z[P]^*[S]^* - Z_{CAC}[C]^*) \tag{32}$$

Finally:

$$\beta\frac{\mathrm{Pe}}{L_N}[C]_x^{*\prime} = D_{CN}[C]_{yy}^{*\prime\prime} + \mathrm{Da}(Z[P]^*[S]^* - Z_{CAC}[C]^* \tag{33}$$

All the dimensionless numbers are marked red.

The final dimensionless system of equations, which will model an P + S ↔ C reaction in a microchannel:

$$\begin{cases} \beta\frac{Pe}{L_N}[P]^{*\prime}_{x} = [P]^{*\prime\prime}_{yy} - Da(Z[P]^*[S]^* - Z_{CAC}[C]^*) \\ \beta\frac{Pe}{L_N}[S]^{*\prime}_{x} = D_N[S]^{*\prime\prime}_{yy} - Da([P]^*[S]^* + \frac{Z_{CAC}}{Z}[C]^* \\ \beta\frac{Pe}{L_N}[C]^{*\prime}_{x} = D_{CN}[C]^{*\prime\prime}_{yy} + Da(Z[P]^*[S]^* - Z_{CAC}[C]^* \end{cases} \quad (34)$$

This system is non-dimensional and considers the parabolic flow profile.

Fundamental physical quantities defining this system of equations are meters, seconds, and mols (three totally). This system depends on the following variables: flowrate U, channel length L, channel width W, concentrations of reagents $[P]^0$ and $[S]^0$, diffusion coefficients of reaction species D_P, D_S and D_C, and finally direct (k_f) and reverse (k_r) rate constants of the complexation reaction (10 totally).

According to the Buckingham – Pi theorem [1], this system will depend on 10 – 3 = 7 dimensionless parameters that naturally appear in the dimensionless version of the equations: Pe, L_N, Da, D_N, D_{CN}, Z, and Z_{CAC}.

Boundary conditions in the dimensionless form:

$$\begin{cases} [P]^{*\prime}_{y}(y = 0) = 0 \\ [P]^{*\prime}_{y}(y = 1) = 0^{\cdot} \end{cases} \quad (35)$$

same for the surfactant S and complex C.

$$\begin{cases} [P]^*(x^* = 0, y^*) = \begin{cases} 1, y^* \le y_r^* \\ 0, y^* > y_r^* \end{cases} \\ [S]^*(x^* = 0, y^*) = \begin{cases} 0, y^* < y_r^* \\ 1, y^* \ge y_r^* \end{cases} \\ [C]^*(x^* = 0, y^*) = 0 \end{cases} \quad (36)$$

Where $y_r^* = \frac{y_r}{w}$

S3.Model for the Ψ-type Chip Geometry

We will proceed to the analysis of the microchip with 3 inputs and the central buffer flow shown in Fig. S2:

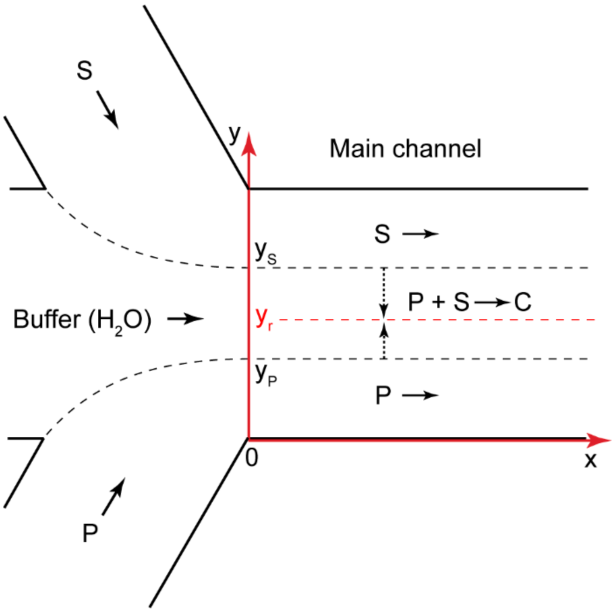

Figure S2. Ψ-type microfluidic chip. P – polyelectrolyte, S – surfactant, C – complex.

Eq. (30) and (31) are applicable to this microchip geometry. We need to re-define the boundary conditions at the junction of inputs (y = 0).

$$\begin{cases} [P](x=0,y) = \begin{cases} [P]^0, y \le y_P \\ 0, y > y_P \end{cases} \\ [S](x=0,y) = \begin{cases} 0, y < y_S \\ [S]^0, y \ge y_S \end{cases} \\ [C](x=0,y) = 0 \end{cases} \quad (37)$$

It is convenient to modify the boundary conditions (32) by introducing the widths of polymer, surfactant, and solvent flows. The widths of these flows in the main channel after the junction of inputs are proportional to polymer Q_P, surfactant Q_S, and buffer Q_B flowrates, respectively. In turn, the coordinates y_P and y_S also represent polymer and surfactant flow widths.]

Then, the width of the polymer flow:

$$y_P = \frac{Q_P}{Q_P + Q_S + Q_B} W \quad (38)$$

The width of the surfactant flow:

$$W - y_S = \frac{Q_S}{Q_P + Q_S + Q_B} W \quad (39)$$

The width of the buffer flow:

$$y_S - y_P = \frac{Q_B}{Q_P + Q_S + Q_B} W \quad (40)$$

If we keep polymer and surfactant flows symmetrical in our experiments ($y_P = W - y_S$), the coordinates y_P and y_S can be expressed as functions of the buffer flowrate only. Introduce the normalized buffer flow width Q_N, which is the flow fraction occupied by buffer:

$$Q_N = \frac{Q_B}{Q_P + Q_S + Q_B} \tag{41}$$

Then, the widths of polymer and surfactant flows are:

$$y_P = W - y_S = \frac{1 - Q_N}{2} W \tag{42}$$

The y_S coordinate:

$$y_S = \frac{1 + Q_N}{2} W \tag{43}$$

The boundary conditions (32) are defined now by the normalized buffer flow width only:

$$\begin{cases} [P](x = 0, y) = \begin{cases} [P]^0, y \le \frac{1 - Q_N}{2} W \\ 0, y > \frac{1 - Q_N}{2} W \end{cases} \\ [S](x = 0, y) = \begin{cases} 0, y < \frac{1 + Q_N}{2} W \\ [S]^0, y \ge \frac{1 + Q_N}{2} W \end{cases} \\ [C](x = 0, y) = 0 \end{cases} \tag{44}$$

These boundary conditions in the dimensionless form:

$$\begin{cases} [P]^*(x^* = 0, y^*) = \begin{cases} 1, y^* \le \frac{1 - Q_N}{2} \\ 0, y^* > \frac{1 - Q_N}{2} \end{cases} \\ [S]^*(x^* = 0, y^*) = \begin{cases} 0, y^* < \frac{1 + Q_N}{2} \\ 0, y^* \ge \frac{1 + Q_N}{2} \end{cases} \\ [C]^*(x^* = 0, y^*) = 0 \end{cases} \tag{45}$$

S4.Reaction Initiation Conditions in a Ψ-type Chip

A characteristic diffusion time of the reagents to the site of reaction y_r according to the microchip geometry shown in Fig. S2 can be estimated as follows [1]:

$$t_{in} = \frac{(y_S - y_r)^2}{4D_S} = \frac{(y_r - y_P)^2}{4D_P} = L/U \tag{46}$$

Consider the central segment of this equation:

$$\frac{(y_S - y_r)^2}{4D_S} = \frac{(y_r - y_P)^2}{4D_P} \tag{47}$$

and transform it into the following form:

$$\frac{(y_S - y_R)^2}{(y_r - y_P)^2} = \frac{4D_S}{4D_P} \tag{48}$$

Then:

$$\frac{y_S - y_r}{y_r - y_P} = \sqrt{D_N} \tag{49}$$

and:

$$y_S - y_r = \sqrt{D_N}(y_r - y_P) \tag{50}$$

Therefore:

$$\sqrt{D_N}y_r + y_r = y_S + \sqrt{D_N}y_P \tag{51}$$

and:

$$y_r(1 + \sqrt{D_N}) = y_S + \sqrt{D_N}y_P \tag{52}$$

The coordinates y_P and y_S are the functions of Q_N (Eq. 37 and 38), then:

$$y_r\left(1 + \sqrt{D_N}\right) = \frac{1 + Q_N}{2}W + \sqrt{D_N}\frac{1 - Q_N}{2}W \tag{53}$$

Transform this equation:

$$y_r\left(1 + \sqrt{D_N}\right) = W\frac{1 + Q_N + \sqrt{D_N} - \sqrt{D_N}Q_N}{2} \tag{54}$$

and:

$$y_r\left(1 + \sqrt{D_N}\right) = W\frac{\left(1 + \sqrt{D_N}\right) + Q_N\left(1 - \sqrt{D_N}\right)}{2} \tag{55}$$

Divide Eq. 50 by $\left(1 + \sqrt{D_N}\right)$:

$$y_r = \frac{W}{2}(1 + Q_N\frac{1 - \sqrt{D_N}}{1 + \sqrt{D_N}}) \tag{56}$$

Transform the segment $\frac{(y_R - y_P)^2}{4D_P} = L/U$ of the Eq. 41 by using the Eq. 37 and Eq. 51:

$$\left(\frac{W}{2}(1 + Q_N\frac{1 - \sqrt{D_N}}{1 + \sqrt{D_N}}) - \frac{1 - Q_N}{2}W\right)^2 = 4D_P\frac{L}{U} \tag{57}$$

Divide this equation by W^2:

$$\left(\frac{1}{2}(1 + Q_N\frac{1 - \sqrt{D_N}}{1 + \sqrt{D_N}}) - \frac{1 - Q_N}{2}\right)^2 = 4\frac{D_P}{UW}\frac{L}{W} \tag{58}$$

Continue transformations:

$$\left(\frac{1}{2} + \frac{Q_N}{2}\frac{1 - \sqrt{D_N}}{1 + \sqrt{D_N}} - \frac{1}{2} + \frac{Q_N}{2}\right)^2 = 4\frac{D_P}{UW}\frac{L}{W} \tag{59}$$

then:

$$\left(\frac{Q_N}{2}(\frac{1 - \sqrt{D_N}}{1 + \sqrt{D_N}} + 1)\right)^2 = 4\frac{D_P}{UW}\frac{L}{W} \tag{60}$$

and:

$$\left(\frac{Q_N}{2}\left(\frac{1-\sqrt{D_N}+1+\sqrt{D_N}}{1+\sqrt{D_N}}\right)\right)^2 = 4\frac{D_P}{UW}\frac{L}{W} \quad (61)$$

$$\left(\frac{Q_N}{2}\frac{2}{1+\sqrt{D_N}}\right)^2 = 4\frac{D_P}{UW}\frac{L}{W} \quad (62)$$

Finally:

$$\left(\frac{Q_N}{1+\sqrt{D_N}}\right)^2 = 4\frac{D_P}{UW}\frac{L}{W} \quad (63)$$

The right part of the Eq. (58) is the combination of Peclet number $Pe = UW/D_P$ and the microchannel length-to-with ratio $L_N = L/W$ therefore:

$$\left(\frac{Q_N}{1+\sqrt{D_N}}\right)^2 = 4\frac{L_N}{Pe} \quad (64)$$

Equation 59 predicts the reaction initiation conditions at the output of the microchip with the main channel length L and the inputs of flows as shown in Fig. S2. It is convenient to introduce an integrated dimensionless number $(\frac{Pe}{L_N})_{in}$, which considers competing contributions of microchannel length, width, and flowrates to the reaction initiation in a microchip:

$$(\frac{Pe}{L_N})_{in} = 4(\frac{1+\sqrt{D_N}}{Q_N})^2$$

The $(\frac{Pe}{L_N})_{in}$ parameter depends only on the properties of a specific reaction pair defined by the ratio of diffusivities D_N and the selected microchip operation mode set by solvent flow fraction Q_N. Thus, we propose it as a convenient similarity criterion that estimates the reaction initiation threshold in Ψ-type microchips with arbitrary lengths, widths, and flowrates.

S5.Modeling Reaction Front Positions at Different Rate Constants and Product Diffusion Coefficients

High Damköhler numbers of microfluidic polyelectrolyte-surfactant flows predict that their association reaction is diffusion-controlled and the rate constant is not the governing factor for development of the reaction front.

According to high Peclet numbers, the reaction product (macromolecules with bound surfactant ions) are not likely to diffuse a considerable distance from the reaction front. It is confirmed by numerical simulations performed for a broad range of product diffusion coefficients.

The results of numerical modeling for various rate constants and product diffusion coefficients are summarized in Fig. S3. The points correspond to maximum product concentrations across the microchannel.

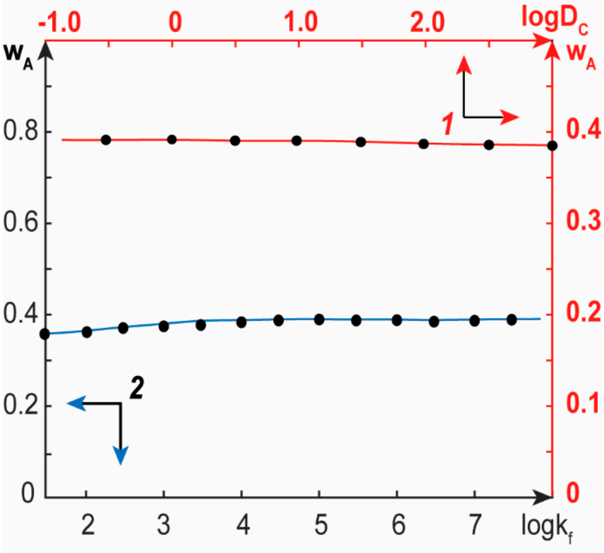

Figure S3. Reaction front positions at different diffusion coefficients of the reaction product D_C (1) and association rate constants k_f (2).

We can see from Fig. S3 that the location of the reaction front depends weakly on the values on the association rate constant and product diffusivity, so these parameters cannot be considered as the governing factors for polyelectrolyte-surfactant association in microfluidic confinement.

S6.Analyzing Precipitation Time Course in Different Flow Modes

Precipitates accumulate in a microchannel with a course of polyelectrolyte-surfactant complexation reaction. The duration of the majority of microfluidic experiments did not exceed 15 minutes that was sufficient to collect samples for the further dynamic light scattering analysis.

To analyze accumulation of precipitates with a course of time, we also performed selected longer microfluidic experiments up to 60 minutes in various operating conditions.

Y-type chips were quickly blocked by precipitates within 5-10 minutes of experiments. We observed no substantial effects of flowrates on the rate of precipitate accumulation in the main channel.

The rate of precipitate accumulation in Ψ-type chips was found to depend on flowrates of the reagents and the solvent and also on the distance from the junction point.

Fig. S4 demonstrates accumulation of precipitates within an hour time in the main channel of a Ψ-type chip. The flowrates of the reagents are selected to provide the conditions $Pe>Pe_{in}$ for this channel length and width.

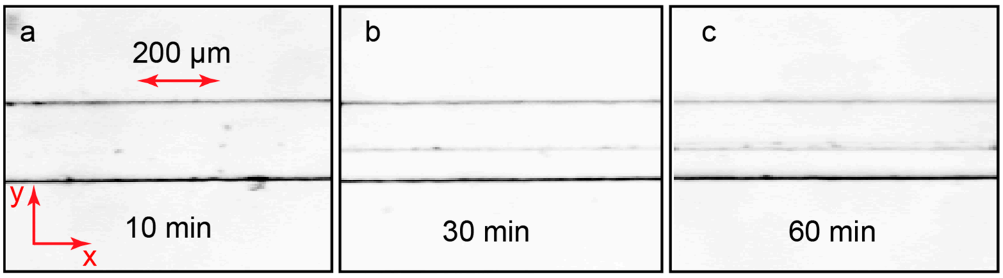

Figure S4. Accumulation of precipitates in the main channel of a Ψ-type chip (L = 15 mm and W = 200 μm). Distance from the junction of inputs: 10 mm. Input flowrates: polymer and surfactant: 10 μl/min, solvent: 40 μl/min.

We can see in Fig. S4 that precipitates accumulated as a thin line after 60 minutes of experiment. Within 10-15 minutes, precipitation is barely detectable by optical microscopy. At such Pe number, the reaction conditions are supposed not to be achieved in this microchip. A minimized precipitation can be associated with formation of tiny amounts of complexes in a 3-5 μm area near microchannel bottom wall due to reduced flowrates in this area and the resulting more intensive transverse diffusion transport of the reagents. Such a minimized precipitation, however, does not affect the chip performance. DLS analysis shows no reliable data on the presence of polymer-surfactant complexes in samples taken from the central output in such conditions.

In the conditions $Pe \approx Pe_{in}$, precipitates accumulate with time as shown in Fig. S5. Precipitates emerge a s straight line (Fig. S5a), then the line grows (Fig. S5b and S5c) to finally obstruct the channel (Fig. S5d). Accumulation of precipitates is more intensive with a course of time if we move closer to the main channel output. The junction point, however, remained clean after 60 minutes of the experiment. A possible reason for such an effect is that $Pe = Pe_{in}$ conditions may not be reached at such short distances form the junction point

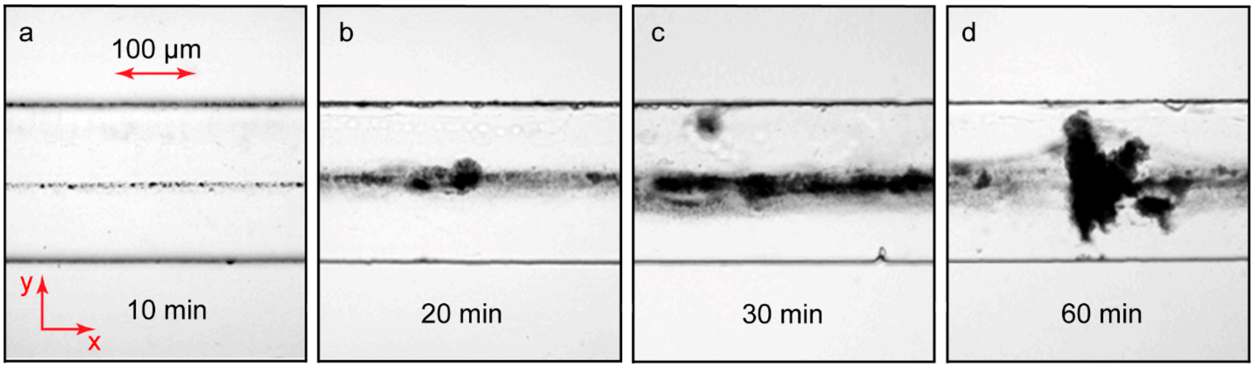

Figure S5. Accumulation of precipitates in the main channel of a Ψ-type chip (L = 15 mm and W = 200 μm). Distance from the junction of inputs: 10 mm. All the input flowrates are 5 μl/min.

In Fig. S6, we demonstrate conditions that correspond to $Pe < Pe_{in}$. Precipitation process is more intensive than one shown in Fig. S5. Obstruction of the main channel output occurs within 10-15 minutes. Precipitation is firstly not-clogging at the junction point shown in Fig. S6a. However, the beginning of the main channel was finally clogged by precipitates in 30 minutes of the experiment (Fig. S6b).

To summarize, in $Pe > Pe_{in}$ conditions, precipitation is minimized. Some traces of precipitates form near microchannel bottom, which do not affect the microchip performance.

In $Pe \approx Pe_{in}$ conditions, precipitation starts near the main channel output and proceeds then to other microchip areas. Microchip remains clean at short distances form the junction point the entire time of the experiments (60 minutes)

In $Pe < Pe_{in}$ conditions, microchip is quickly clogged by precipitates at the output. Precipitation clogs the entire main channel in 30-60 minutes.

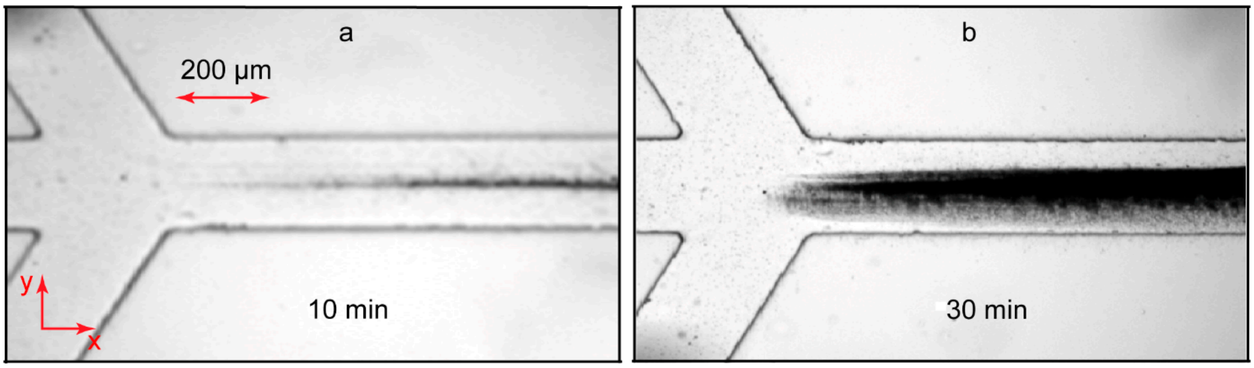

Figure S6. Accumulation of precipitates in the beginning of main channel of a Ψ-type chip (L = 15 mm and W = 200 μm). All the input flowrates are 1 μl/min.

The analysis of the time course of precipitation demonstrates that the accumulation rate of precipitates is proportional to the product concentration in the main channel, which can be predicted by numerical simulations results. Thus, we can predict precipitation conditions in microfluidic devices with our numerical model and control phase separation processes along the main channel with a course of time.

S7.The Matlab Script for Convection-Diffusion-Reaction Equation of Polymer-Surfactant Association in a Microfluidic Channel

```
%THE CHANNEL INCLUDES THREE INPUTS: LEFT - A POLYMER SOLUTION, MIDDLE - WATER,
RIGHT - A MICELLAR SURFACTANT SOLUTION:

%pdex4_Polymer_surf2_1
%First set global variables to be used by all the functions in this script:
global W H L Q_pol Q_solv Q_surf Dpol Dmol Dcompl Cpol Csurf kaf kab U k p Z;

%Input the solutions accuracy (100-10000):

acc=1000;

k = [1;1;1];
p = [1;2;3];
%INPUT THE VALUES, WHICH CHARACTERIZE THE MICROFLUIDIC SYSTEM AND THE POLYMER-
SURFACTANT SOLUTION:

% Input channel width W, µm; channel height, H, µm; and channel length, L, mm:
W=200; H=100; L=15;
%Input the flowrates of polymer Q_pol, solvent Q2 and surfactant Q3, µl/min:
Q_pol = 10; Q_solv = 10; Q_surf = 10;

%Input the diffusion coefficients of polymer, molecular surfactant Dmol,
%micellar surfactant Dmic, and a polymer-surfactant complex Dcompl µm^2/s:
Dpol = 49; Dmol=454; Dcompl = 5;

%Input initial concentration of polymer Cpol, surfactant Csurf and CMC of sur-
factant, mmol/l:
Cpol=6.1; Csurf=6.1;

Z = Csurf/Cpol;

%Input polymer-surfactant association  and dissociation reaction rate constants
kaf l/mol*s and kab, s^-1:
kaf = 30000; kab = 0.0267;

%Calculate the flow velocity, mm/s:
U=(Q_pol+Q_solv+Q_surf)/(W*H)*10^6/60;
% Pe = 1./l*(Q_pol+Q_solv+Q_surf)/Dpol;

%Define  the system of the convection-diffusion reaction equations: three sec-
ond order
%partial differential equations with the source - sink terms (reactions)
%for molecular and micellar surfactant:
a=1;
b=1;
m = 0;

x = linspace(0,a,acc);
```

```
l = linspace(0,b,acc);

sol = pdepe(m,@pdex4pde,@pdex4ic,@pdex4bc,x,l);
%Polymer:
u1 = sol(:,:,1);
%Molecular Surfactant:
u2 = sol(:,:,2);
%Complex:
u3 = sol(:,:,3);

%Plot concentrations of reactants and products, mmol/l
figure
plot(x,u1(end,:),x,u2(end,:),x,u3(end,:));


figure
surf(u3(:,:))
shading interp
% hcb=colorbar;
% hcb.Location = 'south';
% set(hcb,'YTick',[])
colormap(jet)
view(90,-90)
%axis equal
axis off

% ---------------------------------------------------------------
%Define coefficients of the convection-diffusion-reaction equation from the
%dimensionless numbers:
function [c,f,s] = pdex4pde(~,~,u,DuDx)
global W L Dpol Dmol  Dcompl kaf kab U Z Cpol;
%Input the Poiseuille flow parabolic profile conditions:
%Pois = 2*(1-(2.*x-1)^2);
%Introduce the common coefficient for convection components in all the four
equations with the use of global k:

%Input source-sink terms for each equation from rate laws:
s1 = -kaf*Cpol*W*W*Z*0.001.*u(1).*u(2) + kab*W*W.*u(3);
s2 = -kaf*Cpol*W*W*0.001.*u(1).*u(2)+ kab/Z*W*W.*u(3);
s3 = kaf*W*W*Z*0.001*Cpol.*u(1).*u(2) - kab*W*W.*u(3);
%finally set the coefficients for all the equations:
%convection term:
c = [1;1;1].*U*W*W/L;
%diffusion term:
f = [Dpol; Dmol; Dcompl].*DuDx;
%reaction term:
s = [s1; s2; s3];
end

% Set initial conditions:
function u0 = pdex4ic(x)
global Q_pol Q_solv Q_surf
left = Q_pol/(Q_pol+Q_solv+Q_surf);
right = (Q_pol+Q_solv)/(Q_pol+Q_solv+Q_surf);
u0 = [x<=left; x>=right; 0];
end
```

```
% Set boundary conditions:
function [pl,ql,pr,qr] = pdex4bc(~,ul,~,ur,~)
global k p
pl = ul(p);
ql = k;
pr = ur(p);
qr = k;
end
```

References

1. Berthier, J.; Silberzan, P., Microfluidics for Biotechnology, Second Edition. Artech House: London, 2009; p 512.
2. Tabeling, P., Introduction to Microfluidics. Oxford University Press: 2005; p 312.
